# Risk factors for progression of Urolith Associated with Obstructive Urosepsis to severe sepsis or septic shock

**DOI:** 10.1186/s12894-022-00988-8

**Published:** 2022-03-28

**Authors:** J. D. Cao, Z. C. Wang, Y. L. Wang, H. C. Li, C. M. Gu, Z. G. Bai, Z. Q. Chen, S. S. Wang, S. T. Xiang

**Affiliations:** 1 Department of Urology, The Second Affiliated Hospital of Guangzhou University of Chinese Medicine, Fangcun Branch, 36 Chong’an Street, North Dongjiao Road, Guangzhou, 510370 China; 2 Department of Urology, The Second Affiliated Hospital of Guangzhou University of Chinese Medicine, 261 Da Tong Road, Guangzhou, 510120 China; 3 From Department of Andrology, The Second Affiliated Hospital of Guangzhou University of Chinese Medicine, Zhuhai, China; 4 From Department of Nephrology, The Second Affiliated Hospital of Guangzhou University of Chinese Medicine, Guangzhou, China

**Keywords:** Urolith, Urosepsis, Severe sepsis, Septic shock, Risk factors

## Abstract

**Introduction:**

To analyze the risk factors for progression of urolith associated with obstructive urosepsis to severe sepsis or septic shock, we had done the retrospective cross-sectional study, which would facilitate the early identification of high-risk patients.

**Materials and methods:**

Datas were retrospectively reviewed from 160 patients, suffering from obstructive urosepsis associated with urolith between December 2013 and December 2019. There were 49 patients complicating by severe sepsis (severe sepsis group), 12 patients complicating by septic shock (septic shock group), and 99 patients without progressing to severe sepsis or septic shock (sepsis group). The data covered age, gender, BMI (body mass index), time interval from ED (emergency department) to admission, WBC count (white blood cell count), NLR (neutrophil/lymphocyte ratio), HGB (hemoglobin), etc. Datas were analyzed by univariate analyses and multivariate logistic regression analysis. The corresponding nomogram prediction model was drawn according to the regression coefficients.

**Results:**

Univariate analysis showed that the differences of age, the time interval from ED to admission, history of diabetes mellitus, history of CKI (chronic kidney disease), NLR, HGB, platelet count, TBil (total bilirubin), SCr (serum creatinine), ALB (albumin), PT (prothrombin time), APTT (activated partial thromboplastin time), INR (international normalized ratio), PCT (procalcitonin), and positive rate of pathogens in blood culture were statistically significant (P < 0.05). Multivariatelogistic regression analysis showed that age, SCr, and history of CKI were independent risk factors for progression to severe sepsis, or septic shock (P < 0.05).

**Conclusions:**

Aged ≥ 65 years, SCr ≥ 248 mol/L, and history of CKI were independent risk factors for progression of urolith associated with obstructive urosepsis to severe sepsis or septic shock. We need to pay more attention to these aspects, when coming across the patients with urolithic sepsis.

**Supplementary Information:**

The online version contains supplementary material available at 10.1186/s12894-022-00988-8.

## Introduction

Urosepsis refers to the systemic inflammatory response caused by urinary tract infection, whose onset and progression is rapid. People pay more and more attention to urosepsis, because the fatality rate is as high as 28.3–41.1% once it develops into severe sepsis and septic shock [1, 2]. Hoffmann et al. [3] found that Seventy-eight percents patients with urosepsis were caused by obstructive urolith. The research on urosepsis mainly focuses on the risk factors of urosepsis after minimally invasive endoscopic lithotripsy [4, 5], whereas reports on the pre-hospital risk factors for disease progression from initial diagnosis to more severe conditions are sparse. We analyzed the clinical data in a retrospective cross-sectional study and aimed to evaluate the risk factors for the progression of urolith associated with obstructive urosepsis to severe sepsis or septic shock, so as to provide insight on early identification of high-risk patients and better clinical outcomes.

## Materials and methods

A retrospective cross-sectional study was made to analyse the data collected from 160 patients suffering from urolithic sepsis between December 2013 and December 2019 in three branches of Guangdong Provincial Hospital of Traditional Chinese Medicine. The diagnostic criteria for urosepsis [6] are as follows: (1) the clinical symptoms caused by urinary tract infection; (2) the systemic inflammatory response syndrome (must fulfill at least two of the following criteria: ① fever > 38 °C or hypothermia < 36 °C;② tachycardia > 90 beats/minute; ③ tachypnea > 20 breaths/minute or the PCO2 of arterial blood was < 32 mmHg (4.3 kPa); ④ leukocytosis > 12 × 10^9^ /L or leukopenia < 4 × 10^9^ /L, or the ratio of immature white blood cells are ≥ 10%). 160 patientswere divided into sepsis group (99 cases), severe sepsis group (49 cases), and septic shock group (12 cases). For the definition of severe sepsis and septic shock, refer to the Surviving Sepsis Campaign: International Guidelines for Management of Sepsis and Septic Shock: 2016 (2016 SCC Guidelines). Severe sepsis is sepsis with consequent organ dysfunction and/or tissue insufficiency, one of the following: (1) Hypotension caused by sepsis; (2) Lactic acid was greater than normal; (3) Even with adequate fluid resuscitation, urine volume remains < 0.5 ml/kg/h at least 2 h; (4) Non-pneumonia-induced acute lung injury with PaO2/FiO2 < 250 mmHg. (5) Acute lung injury caused by pneumonia with PaO2/FiO2 < 200 mmHg. (6) SCr > 176.8 μmol/L (2.0 mg/ dL); (7) TBil > 34.2 μmol/L (2 mg/dL); (8) PLT < 100 000 mu l; (9) Coagulation disorders (INR > 1.5). Septic shock was defifined as a systolic arterial pressure below 90 mmHg, a mean arterial pressure < 60 mmHg, or a reduction in systolic blood pressure > 40 mmHg from baseline, despite adequate fluid replacement or using vasopressors for at least 1 h.

All patients were diagnosed with sepsis associated with urolith obstructive at admission by attending physician on duty. And data were collected by two senior specialists.Before that,every person had accepted the imaging results, such as urinary system B ultrasound, excretory urogram, or abdominopelvic CT (computed tomography).And all were diagnosed with urinary calculi.Urine and blood sampling was tested within 6–8 h after hospitalization. These patients were accepted Anti-infective therapy, necessary fluid resuscitation and underwent emergency drainage of the upper urinary tract for urolith associated with obstructive urosepsis.We analyzed the following indicators of the patients: age, gender, BMI, history of diabetes, history of CKI, history of anemia, history of hypertension, history of heart disease, history of cancer, history of urolithiasis surgery, the time interval from ED to admission, the highest temperature level, surface area of stone, stone locations, degree of hydronephrosis, WBC count, NLR, platelet count, HBG, ALB, SCr, TBil, PT, APTT, INR, CRP (C-reactive protein), PCT, positive rate of pathogens in urine culture, positive rate of urinary nitrite, urinary leukocyte count, and positive rate of pathogens in blood culture.

The data were analyzed by SPSS 21.0 statistical software. Univariate regression analysis was used to identify statistically significant variables. The F tests was used for the comparison between a variety of measurement data in univariate analysis, and P < 0.05 means a statistically significant difference.Multivariate regression analysis was used to identify independent risk factors. T test is used for the comparison between two groups of measurement data in Multivariate regression. And P < 0.05 means a statistically significant difference.

All methods were carried out in accordance with relevant guidelines and regulations. And all experimental protocols were approved by Ethics Committee of Guangdong Hospital of Chinese Medicine. All patients were informed and consented to this study, and that subjects are under 18, consent was obtained from a parent and/or legal guardian.

## Results

A total of 160 patients met the inclusion criteria. The average BMI was 24.29 kg/m^2^ (range 16.4–37.6 kg/m [2]). Gender distribution was 41 male and 119 female, aged 20‒92 years (median 63 years). General underlyingdiseases included 34 diabetes, 41 CKIs, 30 anemias, 69 hypertensions, 11 heart diseases, 7 cancers and 46 urolithiasis surgeries. The average time interval from ED to admission was 9.66 h (range 1–48 h).

There were significant differences among the sepsis group, the severe sepsis group and the septic shock group in terms of NLR (P = 0.005), platelet count (P = 0.000), HBG (P = 0.101), ALB (P = 0.000), SCr (P = 0.000), Tbil (P = 0.004), PT (P = 0.000), APTT (P = 0.000), INR (P = 0.000), PCT (P = 0.000), and positive rate of pathogens in blood culture (P = 0.001). The complete set of results includingthe highest temperature level, WBC count, CRP etc., is listed below (Table 1). Multivariate logistic regression analysis dentified age (P = 0.024), Scr (P = 0.000), and history of CKI (P = 0.010) were independent risk factors for progression of urolith associated with obstructive urosepsis to severe sepsis or septic shock (Table 2).

**Table 1:** Clinical characteristics of 160 patients with urolith associated with obstructive urosepsis

Variable	Sepsis group	Severe sepsis group	Septic shock group	Pvalue
Gender (n, %)				
Male Female	22 (22.2) 77 (77.8)	34 (69.4) 15 (30.6)	4 (33.3) 8 (66.7)	0.206
Age(years)	55.38 ± 15.45	65.04 ± 12.88	71.83 ± 11.27	0.000
BMI (kg/m^2^)	24.01 ± 3.55	24.69 ± 4.69	23.45 ± 3.14	0.599
History of diabetes(n, %)				
Yes No	15 (15.2) 84 (84.8)	12 (24.5) 37 (75.5)	7 (58.3) 5 (41.7)	0.003
History of CKI (n, %)				
Yes No	17 (17.2) 82 (82.8)	18 (36.7) 31 (63.3)	6 (50.0) 6 (50.0)	0.001
History of anemia (n, %)				
Yes No	15 (15.2) 84 (84.8)	12 (24.5) 37 (75.5)	3 (25.0) 9 (75.0)	0.148
History of hypertension (n, %)				
Yes No	39(39.4) 60(60.6)	25(51.0) 24(49.0)	5(41.7) 7(58.3)	0.282
History of heart disease(n, %)				
Yes No	2(2.0) 97(98.0)	7(14.3) 42(85.7)	2(16.7) 10(83.3)	0.129
History of cancer (n, %)				
Yes No	4(4.0) 95(96.0)	3(6.1) 46(93.9)	0(0.0) 12(100.0)	0.657
History of urolithiasis surgery (n, %)				
Yes No	28(28.3) 71(71.7)	17(34.7) 32(65.3)	1(8.3) 11(91.7)	0.860
Time interval from ED to admission (h)	8.78 ± 11.92	10.29 ± 11.26	13.92 ± 12.63	0.038
Highest temperature level (°C)	39.05 ± 0.57	39.18 ± 0.74	39.29 ± 0.62	0.130
Surface area of stones (cm^2^)	0.79 ± 1.47	0.51 ± 0.41	0.45 ± 0.45	0.102
Laterality of stones (n, %)				
Left Right	37 (37.4) 62 (62.6)	28 (57.1) 21 (42.9)	3(25.0) 9 (75.0)	0.213
Location of stones (n, %)				
Upper Middle Lower	54 (54.5) 14 (14.1) 31 (31.3)	30 (61.2) 3(6.1) 16(32.7)	4(33.3) 2 (16.7) 6 (50.0)	0.537
Degree of hydronephrosis (n, %)				
Mild Moderate Severe	63 (63.6) 24 (24.2) 12 (12.1)	27 (55.1) 15 (30.6) 7 (14.3)	9 (75.0) 3 (25.0) 0 (0)	0.823
WBC count (/L)	18.09 ± 5.93	17.97 ± 8.58	19.11 ± 8.45	0.857
NLR (%)	18.39 ± 12.63	25.74 ± 22.20	31.52 ± 30.32	0.005
Platelet count (10^9^/L)	228.18 ± 73.98	150.73 ± 89.07	113.50 ± 99.97	0.000
HBG (g/L)	121.33 ± 16.92	114.04 ± 18.96	104.42 ± 16.27	0.101
ALB (g/L)	138.94 ± 4.94	34.18 ± 5.26	32.60 ± 5.99	0.000
SCr (μmol/L)	103.15 ± 29.80	248.08 ± 130.71	498.08 ± 660.27	0.000
TBil (μmol/L)	13.58 ± 6.59	18.38 ± 18.10	29.61 ± 39.69	0.004
PT (s)	12.77 ± 1.19	13.47 ± 1.48	15.32 ± 3.33	0.000
APTT (s)	28.83 ± 4.51	30.42 ± 5.32	39.96 ± 14.75	0.000
INR (R)	1.09 ± 0.11	1.17 ± 1.38	1.33 ± 0.29	0.000
CRP (mg/L)	120.64 ± 76.38	156.25 ± 115.68	216.89 ± 108.08	0.148
PCT (ng/mL)	13.51 ± 22.50	40.40 ± 38.57	62.94 ± 45.28	0.000
Positive rate of pathogens in urine culture (n, %)	38(40.9)	19(38.8)	6(50.0)	0.373
Positive rate of urinary nitrite (n, %)	18(29.5)	16(32.7)	16(32.7)	0.665
Urinary leukocyte count (/ul)	2499.50 ± 8818.60	1600.55 ± 6501.60	3789.90 ± 7121.70	0.828
Positive rate of pathogens in blood culture (n, %)	11(22.4)	9(36.0)	7(63.6)	0.001

**Table 2:** Multivariate logistic regression analysis results

Variable	Β	OR	95% CI	P Value
Age	0.045	1.046	1.006 ~ 1.089	0.024
History of diabetes	− 0.157	0.855	0.241 ~ 3.031	0.808
History of CKI	− 1.427	0.24	0.081 ~ 0.711	0.010
Time interval from ED to admission	0.024	1.024	0.970 ~ 1.082	0.386
NLR	− 0.013	0.987	0.959 ~ 1.017	0.395
Platelet count	− 0.004	0.996	0.989 ~ 1.003	0.254
HBG	− 0.010	0.990	0.961 ~ 1.020	0.516
ALB	0.000	1.000	0.891 ~ 1.122	0.996
SCr	0.014	1.014	1.001 ~ 1.020	0.000
Tbil	0.029	1.029	0.989 ~ 1.070	0.151
PT	− 0.253	0.776	0.238 ~ 2.532	0.675
APTT	0.027	1.027	0.913 ~ 1.156	0.653
IN	6.682	797.913	0.002 ~ 280,196,023.5	0.305
PCT	0.008	1.008	0.990 ~ 1.025	0.387

In this study, the collection rates of urine culture (143/160) and blood culture (85/160) were both low. Sixty-three (44.06%) cases (143 bacteria) were positive for bacteria in urine culture, and twenty-five (29.41%) cases (85 bacteria) were positive for bacteria in blood culture.As to the causative bacteria in bacteremia, *Escherichia coli* was the most often isolated (urine culture: 41 strains, 65.08%; blood culture: 22 strains, 81.48%) and gram-negative bacteria occurred in the the majority (urine culture:84.13%; blood culture:96.29%) of our cases(Table 3). All of these patients underwent emergency surgery, including 5 percutaneous nephrostomy and 155 retrograde ureteral stenting.

**Table 3:** Constituent ratios of the pathogens (%)

Pathogens in urine culture	Strains	Constituent ratios (%)	Pathogens in blood culture	Strains	Constituent ratios (%)
Gram-negative bacteria	53	84.13	Gram-negative bacteria	26	96.29
*Escherichia coli*	41	65.08	*Escherichia coli*	22	81.48
*Klebsiella pneumoniae*	2	3.17	*Klebsiella pneumoniae*	3	11.10
*Proteus mirabilis*	2	3.17	*Proteus mirabilis*	1	3.71
*Pseudomonas aeruginosa*	2	3.17	*Pseudomonas aeruginosa*		
*Citrobacter koseri*	2	3.17			
*Morganella morganii*	1	1.58			
*Haemophilus influenzae*	1	1.58			
*Gram-positive bacteria*	7	11.12	Gram-positive bacteria	0	0
*Staphylococcus aureus*	3	4.77			
*Enterococcus faecalis*	3	4.77			
*Streptococcus agalactiae*	1	1.57			
*Fungi*	3	4.75	Fungi	1	3.71
*Candida albicans*	2	3.17	*Candida tropicalis*	1	3.71
*Candida tropicalis*	1	1.58			

**Fig. 1:**
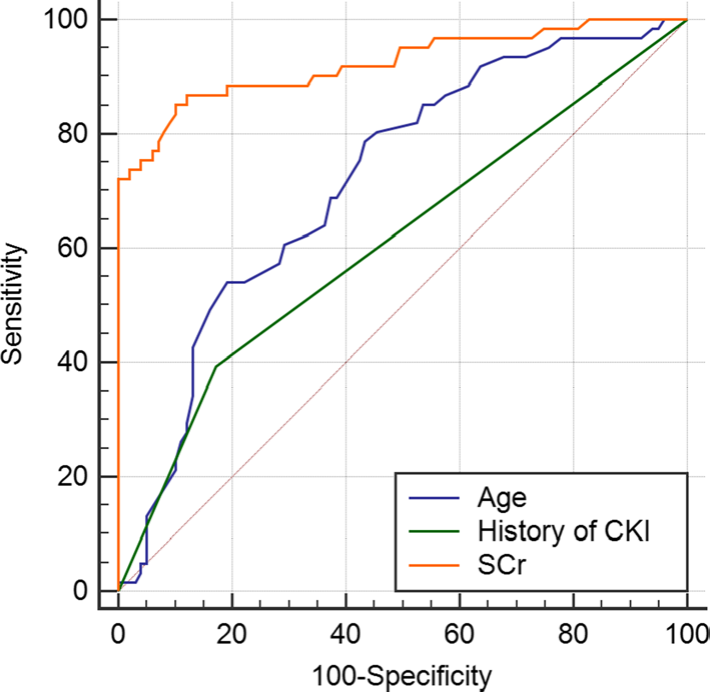
Age, Scr, and history of CKI in predicting the progression of urolith associated with obstructive urosepsis to severe sepsis or septic shock by ROC curve

Based on the logistic multivariate regression analysis, the three independent risk factors (Age, Scr, and history of CKI) were brought into in predicting the progression of urolith associated with obstructive urosepsis to severe sepsis or septic shock by ROC curve. We establish an individualized nomogram prediction model of pre-hospital patients with urolith associated with obstructive urosepsis (Fig. 1).

## Discussion

Urolith associated with obstructive urosepsis is one of the most common emergencies encountered in urology, which is characterized by its severity and rapid progression. Of the 160 pre-hospital patients with urolith associated with obstructive urosepsis in this study, 61 patients developed severe sepsis, or septic shock, whose incidence reaches to 38.13 percents. So it is particularly important and necessary for clinicians to identify highly risk factors for its progression to severe sepsis, or septic shock.

Urolith associated with obstructive urosepsis occurs predominantly in female patients, [7] which also holds true in our study in that the male to female ratio was 1:3.5. The reason is considered to be related to the anatomical characteristics of female lower urinary tract, the higher incidence of female lower urinary tract infection, and the refractory infection of drug-resistant bacteria. However, univariate analysis showed that gender was not a significant risk factor for the progression of urolith associated with obstructive urosepsis to severe sepsis or septic shock. The age data of the three groups showed that the average age of the septic shock group was about 71.83 ± 11.27 years old, which was much higher than that of the sepsis group and the severe sepsis group (55.38 ± 15.45 years and 65.04 ± 12.88 years). Martin et al. [8] found that there was a higher mortality rate if septic patients were older and that age was an independent risk factor for mortality of septic patients. Studies have indicated that age is a risk factor for the progression to severe sepsis or septic shock [9]. In patients with CKI, the higher the baseline Scr, the more serious the kidney damages have, the greater imbalances of homeostasis are in patients with sepsis. In addition, it is difficult for antibiotics to reach the collecting system of the affected kidney, which leads to the poor infection control and worse prognosis.

Early and multidisciplinary comprehensive intervention of sepsis are essential to improve the prognosis and reduce the mortality [10, 11]. The 2016 SCC Guidelines emphasizes the concept of "time to antibiotics" and “hour-1 bundle”. In this study, we defined the “time” as the interval between the ED reception and hospital admission for specialized treatment. By comparing the data between the groups, significant differences were found (P = 0.038). The earlier the time of specialized intervention, the lower the probability of disease progression is.

The pathogenesis of sepsis is still unclear, which may be closely related to the secretion of inflammatory mediators, immune dysfunction, endotoxin translocation, and other factors. When sepsis occurs, neutrophils are released from the bone marrow into the peripheral circulation and apoptosis is delayed [12]. Tulzo et al. [13] showed that the apoptosis of peripheral lymphocyte increased by 5 times in patients with septic shock compared to those with sepsis. Overactivated eutrophils and the decrease of lymphocytes will lead to further suppression of immune function. The NLR reflects the balance between the inflammatory level and the immune status of septic patients, which was significant difference among groups in this study (P = 0.005).Of note, it should be pointed out that there was no significant difference in the level of WBC count (P = 0.857). The WBC count were usually significantly increased in severe infecti on, but significant decrease can also occur. In this study cohort, there were five patients with leukopenia, and the differences were still insignificant after the elimination of the five cases (P = 0.284). Leukopenia is one of the important indicators in the diagnosis of sepsis. The five leukopenic patients in our study accounted for 1.01% in sepsis group, and 6.55% in severe sepsis and septic shock group, and the difference was statistically significant (P = 0.001) by the binary logistic regression analysis. It can be concluded thatleukopeniamay be one of the risk factors for the progression to severe sepsis, or septic shock, and should elicit particular clinical attention.

CRP and PCT are widely used in clinical practice as important indicators to reflect the degree of inflammation in the body. Fukashi Yamamichi et al. [14] analysed on the data of 77 patients with urosepsis, suggesting that CRP was the only risk factor for sepsis involving tumor obstructionto progress to septic shock. In contrast to Yamamichi, most scholars believe that PCT is significantly superior to CRP in the diagnostic sensitivity and specificity of severe sepsis and septic shock, and is also superior to CRP in the assessment of the severity and prognosis of the disease. PCT level is directly proportional to the severity of sepsis caused by bacterial infection, which has important diagnostic significance for bacterial infection. In this study, the average levels of CRP and PCT in patients with severe sepsis, or septic shock, were higher than those in the sepsis group, and the differences of PCT was statistically significant in univariate analysis (P = 0.000). However, the results of multivariatelogistic regression analysis showed no statistical significance.The 2016 SSC guidelines also recommend PCT as an adjunctive indicator in sepsis treatment. However, PCT tests have issues with some false positive and false negative results [15], and different pathogens may lead to different up-regulated PCT responses [16]. As concerned by the SCC guidelines, the PCT can only serve as a supplement in clinical assessment due to the complexity of sepsis and septic shock, therefore the recommendation remains cautious.

Sepsis and septic shock are the main causes of AKI (acute kidney injury), and more than 50% of ICU patients with AKI are associated with sepsis [17]. The incidence of AKI in clinically septic patients is as high as 23% [18]. Currently, it is believed that AKI is caused by the toxic effect of soluble inflammatory factors released during inflammatory response [19]. The higher the Scr level, the worse prognosis of patients with urosepsis will have, and it can be used as an independent risk indicator to predict the disease progression and prognosis [20]. Hypoproteinemia is also common complications of sepsis and related to the severity and prognosis of the disease. Hypoproteinemia can be used as an important indicator to evaluate the prognosis of septic patients: the lower the serum albumin, the lower immunity level and the more severe of the condition are [21]. The Japanese study confirmed that the decrease of serum albumin can be used to predict the risk of septic shock in patients with acute obstructive pyelonephritis [22].

The massive release of inflammatory mediators in septic patients activates the coagulation system, which leads to coagulation disturbance and thrombocytopenia [23, 24]. Kamei [25] found that thrombocytopenia was high risk factors for severe infection. Severe sepsis often leads to decreased platelet levels and relevant studies have confirmed that thrombocytopenia can be used as a risk factor for predicting the progression of sepsis [26, 27]. Severe infection, DIC (diffuse intravascular coagulation), cytophagocytosis, and even immunosuppression in patients with sepsis can lead to excessive consumption of platelets, leading to thrombocytopenia, which may independently alter the patient's immune response to infection [28, 29]. If the platelet level can recover rapidly after treatment, it often indicates a good prognosis, which is supported by a study of prognostic factors for septic patients [30].

Pathogen culture and drug sensitivity identification are important clinically for the treatment and prognosis. In this study, the collection rates of urine culture (143/160) and blood culture (85/160) were both low, which raises the concern of standard of care and should be improved in the future. In this study, 63 strains of pathogens were isolated in urine culture and a total of 27 strains of pathogens were isolated in blood culture (Table 3).The positive rate of blood culture, as the "gold standard", is not high, which may be related to early treatment of broad-spectrum antibiotic. It is worth noting that ideally the blood culture specimens should always be collected prior to the administration of antimicrobial therapy. Current studies have shown that compared to gram-positive bacterial infection, patients with gram-negative bacterial infection are more severely afflicted and are more prone to severe sepsis or septic shock. In this study, gram-negative bacteria were the main culprit in the blood stream of patients with urosepsis and the difference in blood culture positive rate among groups was significant (sepsis group: severe sepsis group: septic shock group = 22.44%: 36.00%: 63.63%). Univariate analysis showed that positive blood culture was a risk factor for sepsis progression, although its contribution was no longer significant in multivariate logistic analysis.

Multivariate logistic regression analysis was performed on the risk factors selected from the above univariate analysis for sepsis progression. The results showed that the differences in age, Scr and history of CKI were statistically significant (P = 0.024, P = 0.000, P = 0.010, respectively). Older age is one of the independent risk factors for the progression of sepsis. According to our study, if the patients are older than 65 years, their systemic inflammatory responses are more intense. Scr level has been used to evaluate the degree of AKI in patients with urosepsis.Patients with urosepsis associated with urolith obstructive may have rapid deterioration of renal function in a short period of time, especially when SCr ≥ 248 mol/L, which leads to increased serum concentrations of systemic inflammatory mediators and further aggravates the risk of infection. This phenomenon is more obvious in patients with CKI.For septic patients with chronic renal insufficiency, early intervention to protect renal function is helpful to improve the prognosis and prevent disease progr ession. If the patients who progressed to severe sepsis group and septic shock group were grouped into one group, that is the progressive group (61 cases in total); this study, the datas were simply divided into the sepsis group (99 cases) and the progressive group. By comparing the differences in age, Scr, and history of CKI between the two groups, the ROC curve was used to compare the value of these three risk factors in predicting the progression of urolith associated with obstructive urosepsis to severe sepsis or septic shock, respectively. The results showed that age, Scr and history of CKI all had good predictive functions. The area under ROC curve was 0.718, 0.923 and 0.611, respectively, and the prediction accuracy of Scr was higher than the other two datas (Fig. 1).

The main limitation of this study lies in that this is a retrospective cross-sectional study, with deficiencies such as incomplete test panels, ambiguous definition of indicators, and inconsistent treatment regimens, which need to be addressed by a better designed prospective study.

## Conclusions

To summarize our findings from this multicenter retrospective cross-sectional study, patients aged ≥ 65 years, Scr ≥ 248 mol/L, and history of CKI were independent risk factors for progression of urolith associated with obstructive urosepsis to severe sepsis or septic shock. It is essential to halt the progression of urosepsis by identifying its risk factors and to treat it as early as possible.

## Supplementary Information

**Additional file 1.** Author information.

**Additional file 2.** Table legend.

**Additional file 3.** Original data.

## Data Availability

Not applicable.

## Abbreviations

BMI: Body mass index
ED: Emergency department
WBC count: White blood cell counts
NLR: Neutrophil/lymphocyte ratio
HGB: Hemoglobin
CKI: Chronic kidney disease
Tbil: Total bilirubin
SCr: Serum creatinine
ALB: Albumin
PT: Prothrombin time
APTT: Activated partial thromboplastin time
INR: International normalized ratio
PCT: Procalcitonin
CRP: C-reactive protein
DIC: Diffuse intravascular coagulation
AKI: Acute kidney injury
