# Supplementary material for: Risk factors for progression of Urolith Associated with Obstructive Urosepsis to severe sepsis or septic shock
Source: BMC Urol. 2022 Mar 28;22:46. doi: 10.1186/s12894-022-00988-8 (PMC8962082; doi:10.1186/s12894-022-00988-8)
Supplement: Supplementary file 1 — Additional file 1. Author information. [file 12894_2022_988_MOESM1_ESM.docx]

**Risk factors for progression of Urolith Associated with Obstructive Urosepsis to severe sepsis or septic shock**

Cao JD, Wang ZC, Wang YL, Li HC, Gu CM, Bai ZG, Chen ZQ, Wang SS

and Xiang ST

^Author information^

*Department of Urology, The Second Affiliated Hospital of Guangzhou University of Chinese Medicine, Guangzhou, 510120，China.*

*Jiadong Cao，Zhichao Wang，Chiming Gu，Zunguang Bai，Zhiqiang Chen，Shusheng Wang，Songtao Xiang*

*（JD Cao，Email：[121587061@qq.com；ZC](mailto:121587061@qq.com；ZC) Wang：[13602495696@163.com](mailto:13602495696@163.com)；CM Gu：aqf600@163.com ；*

*ZQ Chen：[zhi57@163.com；SS](mailto:zhi57@163.com；SS) Wang：* [*13503059270@163.com；ST*](mailto:13503059270@163.com；ST) *xiang：[tonyxst@163.com](mailto:tonyxst@163.com)）*

*Department of Andrology, The Second Affiliated Hospital of Guangzhou University of Chinese Medicine, Zhuhai, 519015，China.*

*Youlian Wang，Email：wyl1767@126.com*

*Department of Nephrology, The Second Affiliated Hospital of Guangzhou University of Chinese Medicine, Guangzhou, 510120，China*

*Hucai Li ，Email：1014584813@qq.com*
